# Photooxidation of Nonanoic Acid by Molecular and Complex Environmental Photosensitizers

**DOI:** 10.1021/acs.jpca.4c05608

**Published:** 2024-11-05

**Authors:** Grace Freeman-Gallant, Emily J. Davis, Elizabeth Scholer, Onita Alija, Juan G. Navea

**Affiliations:** Chemistry Department, Skidmore College, Saratoga Springs, New York 12866-1632, United States

## Abstract

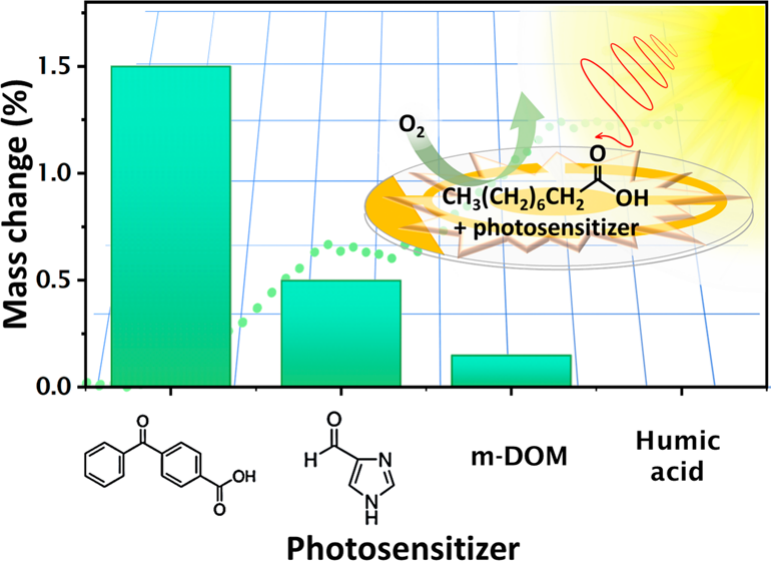

Photochemical aging and photooxidation of atmospheric particles play a crucial role in both the chemical and physical processes occurring in the troposphere. In particular, the presence of organic chromophores within atmospheric aerosols can trigger photosensitized oxidation that drives the atmospheric processes in these interfaces. However, the light-induced oxidation of the surface of atmospheric aerosols, especially those enriched with organic components, remains poorly understood. Herein, we present a gravimetric and vibrational spectroscopy study aimed to investigate the photosensitized oxidation of nonanoic acid (NA), a model system of fatty acids within organic aerosols, in the presence of complex organic photosensitizers and molecular proxies. Specifically, this study shows a comparative analysis of the photosensitized reactions of thin films containing nonanoic acid and four different organic photosensitizers, namely marine dissolved organic matter (m-DOM) and humic acids (HA) as environmental photosensitizers, and 4-imidazolecarboxaldehyde (4IC) and 4-benzoylbenzoic acid (4BBA) as molecular proxies. All reactions show predominant photooxidation of nonanoic acid, with important differences in the rate and yield of product formation depending on the photosensitizer. Limited changes were observed in the organic photosensitizer itself. Results show that, among the photosensitizers examined, 4BBA is the most effective in photooxidizing nonanoic acid. Overall, this work underscores the role of chromophores in the photooxidation of organic thin films and the relevance of such reactions on the surface of aerosols in the marine environment.

## 1 Introduction

Light-absorbing organic chromophores are ubiquitous in the terrestrial and marine boundary layer (MBL), where they can act as photosensitizers and are known to initiate daytime chemistry in the environment.^1−6^ These chromophores are found within the marine boundary layer and are known to partition into sea spray aerosols (SSA).^7−9^ As the largest source of natural aerosols, the presence of photosensitizer components within SSA can exert substantial influence on Earth’s atmosphere and climate. Similarly, atmospheric aerosol particles have been found to contain chromophores that resemble terrestrial and aquatic humic and fulvic acids.^10^ The atmospheric impact of these organic chromophores has been linked to aerosol aging, photooxidation, formation of secondary organic aerosol (SOA), changes in the chemical balance of the atmosphere, and aerosol’s ability to act as cloud condensation nuclei (CCN).^3,11−16^ Yet, the extent of photooxidation of these organic complex aerosols in the marine atmosphere remains poorly understood.^7,17,18^

Sea spray aerosols (SSA) are rich in marine organic species, particularly marine dissolved organic matter (m-DOM), which represents one of Earth’s largest carbon reservoirs.^19,20^ A fraction of m-DOM, known as marine chromophoric dissolved organic matter (m-CDOM), absorbs light within the solar spectral region.^2,19−21^ These chromophores have the potential to act as efficient photosensitizers and are believed to initiate photochemistry in the marine boundary layer.^20,21^ Therefore, it is imperative to understand the photochemical reactions induced by m-CDOM at a molecular level. However, m-CDOM is highly complex, consisting of a mixture of aromatic and aliphatic hydrocarbon structures with many functional groups.^21−23^ The terrestrial counterpart of m-CDOM, humic acid (HA), is similarly complex, consisting of a diverse array of organic substances commonly found in fog, cloudwater, and coastal environments.^24^ Given the high complexity of these environmental light-absorbing compounds, molecular mimics are essential for effectively studying photosensitized reactions and gaining molecular-level insights into their processes.

Recent studies have explored how these complex environmental photosensitizers, m-CDOM and HA, react with single components, comparing their effects to those of model systems such as 4-benzoylbenzoic acid (4BBA), a commonly used photosensitizer.^6,20,21,25−27^ In the case of 4BBA, the high aromaticity and low solubility closely mimics some of the physical and chemical properties of m-CDOM and HA.^21^ In addition to these similarities, Alves et al. found that m-CDOM is enriched in nitrogen and can enhance light-initiated chemistry. This suggests that these nitrogen-containing structures within m-CDOM could influence its photosensitization properties in ways not captured by 4BBA.^23^ To better understand the role of nitrogen in photosensitization, imidazole carboxaldehydes have been used to mimic various light-absorbing, atmospherically complex interfaces.^25−26,27^ In this work, we used 4-carboxaldehyde imidazole (4IC) as a nitrogen-containing photosensitizer. Similar imidazole-derived molecules, present in secondary organic aerosols (SOA) formed by the reaction of ammonium salts with α-dicarbonyls,^28^ have recently been used as models for nitrogen-containing atmospheric chromophores.^29−32^

In addition to the chromophoric organic matter found in the atmospheric boundary layer, fatty acids are also found in atmospheric aerosols, in particular throughout the marine environment.^20,33^ These organic fractions are known to have high surface activity and influence the chemistry in the atmosphere through surface driven reactions.^16,34−38^ In this work, we examined the photosensitized oxidation of nonanoic acid (NA), a fatty acid commonly found in the surface of SSA.^39−41^ Photosensitization experiments are carried out through two different environmental photosensitizers (m-CDOM and HA) and two molecular proxies (4BBA and 4IC). The oxidation of thin films containing a mixture of photosensitizer and NA was investigated to simulate surface photochemistry of atmospheric aerosols such as SSA and coastal systems. Two different proportions of fatty acid to photosensitizer, under atmospherically relevant conditions, were investigated in the presence of solar radiation. In addition, this work explores the mechanistic differences between the molecular proxies of photosensitizers used.^20,27^ Overall, this work reports the kinetics of the light-initiated oxidation reaction of NA, under different photosensitizers and their proxies.

## 2 Experimental Section

### 2.1 Materials

Four thin films of a mixture of fatty acid with different photosensitizers were examined. Nonanoic acid (NA, Sigma-Aldrich) was used as a proxy of fatty acids within SSA and SSML.^20^ Two commonly used molecular photosensitizer models, 4-benzoylbenzoic acid (4BBA, Sigma-Aldrich) and 4-imidazolecarboxylahyde (4IC, Sigma-Aldrich) were used to prepare two sets of thin films: one with a mass ratio of 1:5 photosensitizer to nonanoic acid, and another with a higher fatty acid content at a mass ratio of 1:10. These two molecular photosensitizers are both aromatic and have carbonyl functional groups, making them appropriate mimics of environmental chromophores. Two complex environmental samples, humic acid (HA, Sigma-Aldrich) and marine dissolved organic matter (m-DOM) were also prepared at the same mass ratios. The m-DOM sample used here was collected from a large-scale mesocosm campaign, the NSF-CAICE 2019 SeaSCAPE.^20^

### 2.2 *In Situ* Flow Reactor

**Figure 1:**
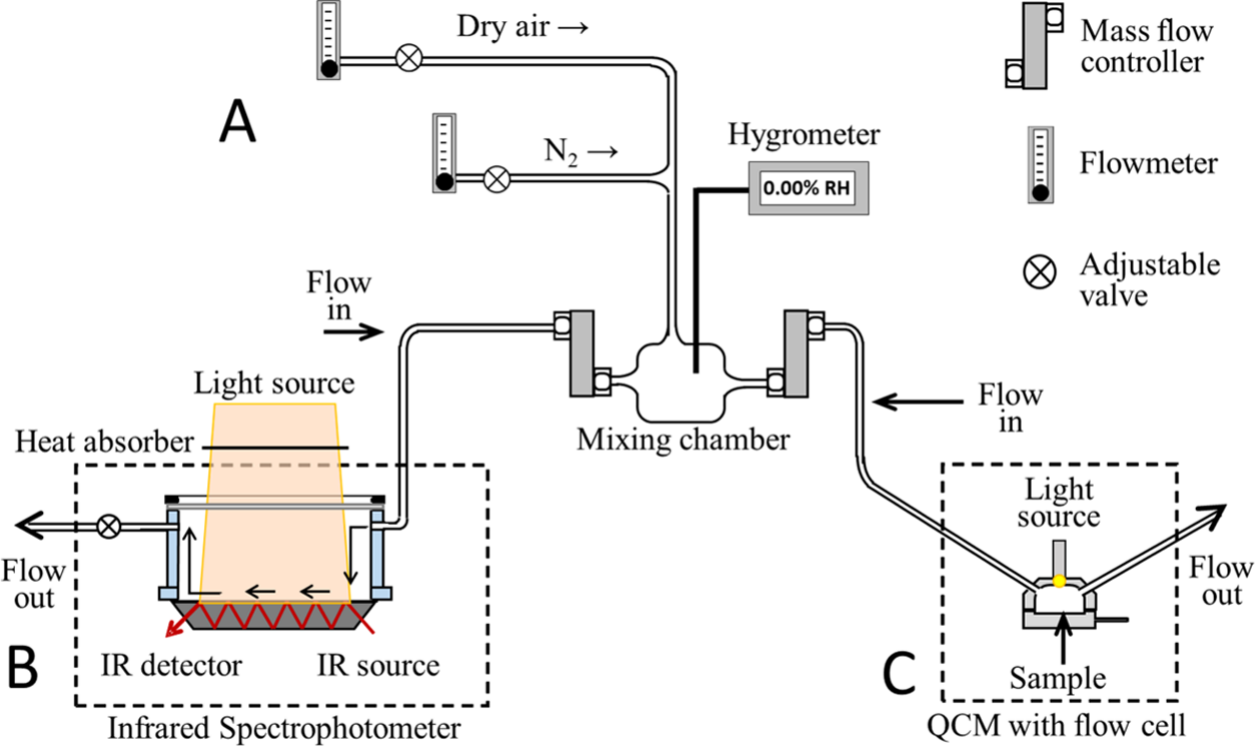
Schematic diagram of the QCM and HATR-FTIR flow system. The experimental apparatus is divided into three segments: (A) Gas control manifold, (B) HATR flow cell with broadband light source in a purged spectrophotometer compartment, and (C) QCM flow chamber with broadband light source in a purged enclosure.

Experiments of the photooxidation of thin films of mixtures containing nonanoic acid and photosensitizers were performed in a tandem gravimetric and vibrational spectroscopy flow system modified from a previously described apparatus.^42^Figure 1 shows the two-dimensional analysis experimental setup: the first section is a quartz crystal microbalance (QCM200, SRS) flow system modified to expose the sample to simulated solar irradiation. The second section is a commercial horizontal attenuated total reflection Fourier transformed infrared spectrophotometer (HATR-FTIR, Thermo) designed to allow solar irradiation of thin films, equivalent to that in the QCM section. The dual system is connected to an air dryer (Balston 75–60) to purge the spectrophotometer compartment and the quartz crystal microbalance (QCM) enclosure.

Both the QCM and IR sections of the experimental setup use corresponding broadband (λ > 300 nm) xenon arc solar simulators (Newport 67005) to irradiate the thin films, with an average output of 130 mW/cm^2^, approximately equivalent to one solar constant. Light sources were positioned above the photochemical cells for the QCM and FTIR flow systems. In the FTIR setup, samples were uniformly deposited in a 7.3 cm × 0.7 cm germanium horizontal attenuated total reflection (HATR) crystal designed for 20 internal reflections (PIKE), enclosed in a custom-made Teflon flow cell with a window on top to allow photochemistry experiments.^3,43,44^ Due to the limited sample quantity, m-DOM FTIR analysis was performed using a 1.5 mm diameter iTR ATR ZnSe crystal (Thermo), also enclosed in a custom-made Teflon flow cell with a window on top to allow solar radiation to reach the sample. In a typical experiment, about 50 mg of visually homogeneous sample was deposited on the ATR crystal. In all FTIR cases, a beam turning mirror assembly (Newport 66245), equipped with a heat absorber window to eliminate infrared radiation and ensure isothermal reaction conditions at 298 K, was used to direct the light to the sample. For the QCM, a focusing assembly (Newport 77776) and a liquid light line (Newport 77628) were used to direct the light to the sample and remove infrared radiation, keeping the temperature constant at 298 K. Films with a photosensitizer to nonanoic acid ratio of 1:5 or 1:10 were uniformly deposited as visually homogeneous mixtures on a 1-in. diameter Au/Cr polished quartz crystal enclosed in a flow cell for QCM analysis. The photosensitizers used in this study included 4BBA, 4IC, m-DOM, and HA, In order to compare these molecular photosensitizers, the absorbance spectra of both 4BBA and 4IC thin films containing NA is shown in SI, Figure S1.

All FTIR measurements were conducted *in situ* to qualitatively monitor the oxidation of the sample during irradiation. The Teflon enclosure of HATR crystal, with the sample placed directly on the crystal, is designed to allow a continuous flow of dry air or other gaseous mixtures when coupled to a flow system, as shown in Figure 1. This flow cell was positioned in the purged internal compartment of an FTIR spectrophotometer (Nicolet 6700). Infrared measurements of the photooxidation of the sample films were collected from 900 to 4000 cm^–1^ at 4 cm^–1^ resolution by averaging 100 scans. The QCM segment provides quantitative information to accurately determine the amount of oxygen added to the sample through a gravimetric measure.^45,46^ The QCM measures changes in the frequency of the polished quartz crystal based on its piezoelectric properties. When the mass loading is less than 2% of the unloaded crystal frequency, the thin sample deposited on the crystal is treated as an extension of its surface. Thus, the relationship between the frequency change and the mass change can then be correlated using the Sauerbrey equation^47^
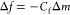
1where Δ*f* represents the change in frequency of the crystal (Hz), Δ*m* denotes the change in mass (μg/cm^2^), and *C*_f_ is the quartz sensitivity factor, which is 56.6 Hz μg^–1^ cm^–2^ for the 5 MHz crystal and remained constant across the sample mass ranges tested. Samples on the QCM ranged from 3 to 5 mg per crystal area. In a typical QCM experiment, the thin film mixture of nonanoic acid and photosensitizers is exposed to alternating 20 min intervals of dark and light, under a flow of 2.5 slpm of dry air or argon, for a total duration of 1 h and 40 min. Similarly, a typical experiment for FTIR was conducted under comparable conditions, with the sample exposed to light for at least 40 min.

### 2.3 *Ex Situ* Analysis of Products

Following exposure to solar radiation in the QCM and the FTIR, postreaction samples containing either 4BBA or 4IC mixed with nonanoic acid were further analyzed using liquid chromatography–mass spectrometry (LCMS, Thermo Vanquish/ISQ-EC) to determine the photooxidation products. Samples containing HA and m-DOM were omitted from this analysis due to the complexity of the photosensitizer (*vide infra*). Analysis of the irradiated samples was carried out using an Ultra AQ C18 column with automatic injections and at a flow rate of 0.250 mL min^–1^, with Chromeleon software package used to assign molecular signatures.

## 3 Results and Discussion

### 3.1 Photooxidation of Nonanoic Acid in the Presence of Molecular Photosensitizers

**Figure 2:**
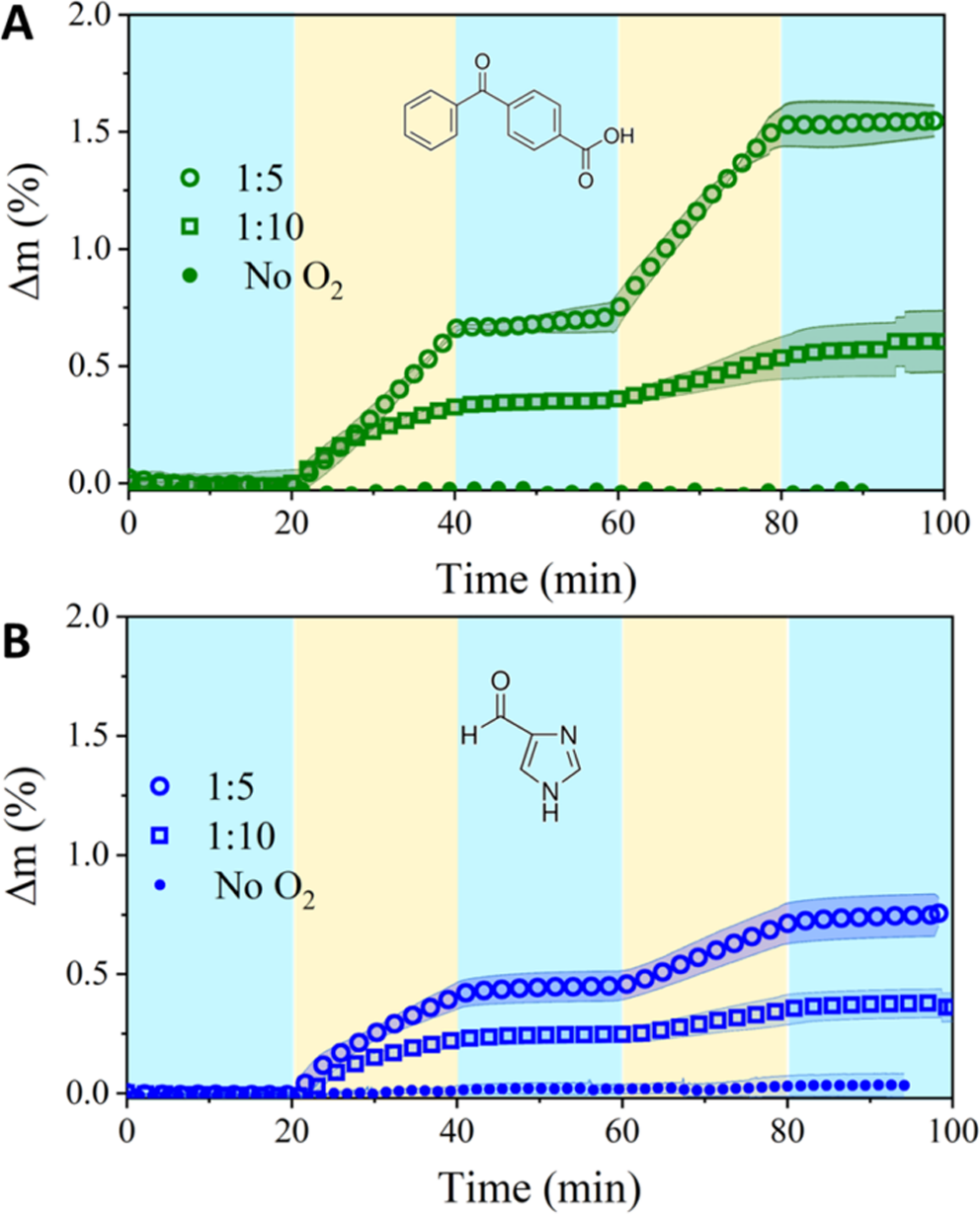
Percentage of mass increase attributed to photoinduced oxidation of thin films with varying mass ratios of photosensitizer to NA (photosensitizer/NA). Two different photosensitizers were used: (A) 4BBA, (B) 4IC. The change in mass labeled “No O_2_” indicates the mass analysis of a 1:5 mixture under anaerobic conditions. Shade represents standard deviation of triplicate experiments. Only 0.1% of data is plotted for clarity.

Gravimetric results of NA mixed with either 4BBA or 4IC under dry air are shown in Figure 2A,B, respectively. The blue shaded sections represent the changes in mass of the thin film samples in the darkness and the yellow shaded sections indicate the samples exposed to solar simulated light. It is clearly observed that mass increases during light cycles, which we interpret as oxygen addition in the samples through photosensitized oxidation (*vide infra*).^20^ Minimal to no mass change was observed for both 4BBA and 4IC samples when irradiated under near-oxygen-free conditions, achieved with a 2.5 slpm flow of ultrahigh purity argon. Neither 4BBA nor 4IC thin films showed any mass increase when irradiated without NA. Finally, irradiation of NA in the absence of either photosensitizers did not result in detectable mass changes.

As shown in Figure 2, there is a higher photoinduced mass increase for mixtures with higher proportion of photosensitizer (1:5 mass ratio of photosensitizer/NA). The rates of photooxidation were extracted from the slopes of a linear fit of the first and second light cycles, with the final rates summarized in Table 1. These rates do not include the possibility of minor simultaneous mass loss through decomposition or fractionation during oxidation reactions, which can result in the formation of oxidized C7 or C8 products. While post irradiation analysis indicates that the fraction of these products is minor, it also suggests that the slopes represent a lower limit of the rate of oxidation, as there is a concurrent small mass loss during the oxygen uptake by NA.

**Table 1:** Photooxidation Rates of Nonanoic Acid by Molecular Photosensitizers 4BBA and 4IC in the Presence of Light and Dry Air^a^

photosensitizer/NA	rate (×10^–5^ mmol O s^–1^)
4BBA/NA 1:5	4.7 ± 0.8
4BBA/NA 1:10	1.4 ± 0.3
4IC/NA 1:5	1.5 ± 0.2
4IC/NA 1:10	0.8 ± 0.2

a Estimated rates assume that all mass changes are the net effect of oxygen reactive uptake.

As the proportion of photosensitizer decreases from a 1:5 to a 1:10 mass ratio with nonanoic acid (NA), the rate of mass increase from oxygen reactive uptake also decreases. This increase in the reaction rate with more photosensitizer suggests that the rate is more dependent on the amount of photosensitizer than on the amount of fatty acid. Comparing the molecular mimics, 4BBA is a more effective photosensitizer, with samples containing 4BBA exhibiting a mass increase of 1.5% for the 1:5 mass ratio and 0.5% for the 1:10 ratio. Correspondingly, thin films containing 4IC as photosensitizer reached a mass increase of 0.6% for the 1:5 samples and 0.3% for the 1:10 samples. This difference may be partly due to the varying optical depths of the 4BBA and 4IC thin films: 4BBA exhibits more intense absorbance bands, while 4IC absorbs lower-energy wavelengths, leading to greater overlap with the solar simulator’s spectral irradiance (Figure S1). The 1:5 sample containing 4BBA had a reaction rate approximately three times faster than that of the 1:10 sample. Conversely, for 4IC, the 1:5 sample had a reaction rate about twice that of the 1:10 sample. These changes in mass gain due to oxygen addition are consistent with similar photosensitizing studies conducted in the aqueous phase, where isomers of imidazole carboxaldehyde exhibit a lower quantum yield than 4BBA, resulting in photooxidation when imidazole was present compared to that of 4BBA.^27^

Overall, for both 4BBA and 4IC, increasing the amount of photosensitizer results in an increased reaction rate. This change from 1:10 to 1:5 mass ratio can also be interpreted as a decrease in the concentration of NA by half. However, the reaction rate does not decrease proportionally; instead, it increases, which is consistent with the reaction rate being more dependent on the amount of photosensitizer than on the concentration of nonanoic acid. The disproportionate increase in mass as the proportion of photosensitizer varies indicates a nonlinear relation between the rate of photooxidation and the amount of photosensitizer.

**Figure 3:**
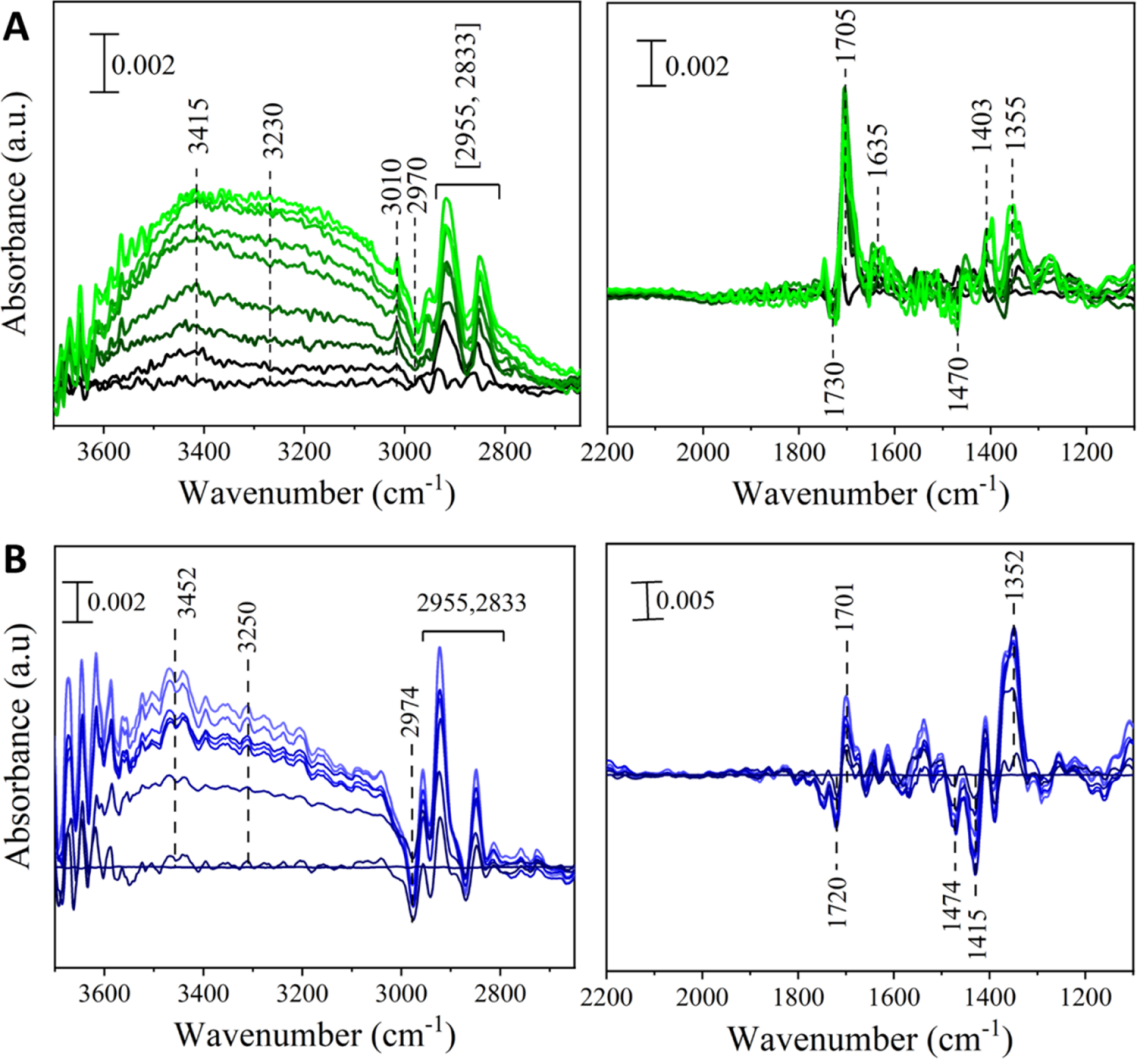
Selected spectra of the ATR–FTIR, referenced to the initial spectrum of NA, in the presence of (A) 4BBA, (B) 4IC. Spectra presented with 10 min intervals for at least 40 min of irradiation. Lines become increasingly light with increased time. No significant absorption features are observed in the region between 2200–2700 cm^–1^.

Vibrational spectroscopy of NA mixed with either photosensitizer under dry air are shown in Figure 3A,B for 4BBA and 4IC, respectively. Thin film mixtures containing either photosensitizers show a significant increase in absorption intensity in the 3000 to 3600 cm^–1^ region relative to the time of irradiation. The broad positive absorption bands at 3415 and 3320 cm^–1^ in Figure 3A and 3452 and 3250 cm^–1^ in Figure 3B, are attributed to the O–H stretching from the formation of oxygenated products.^20^ The growth of positive absorptions bands at 1705 and 1701 cm^–1^ in Figure 3A,B, respectively, are due to carbonyl C=O stretches from the formation of multiple aliphatic ketone and aldehyde species, characteristic of oxidation products.^20,48−50^ The growth of negative absorption bands observed at 1730 in Figure 3A and 1720 cm^–1^ in Figure 3B, while low in intensity, arise from changes in the carboxyl group in the photosensitizer, suggesting that a small proportion of reactions involve changes to the photosensitizer itself. Finally, the growth of the absorption bands at 1403 and 1355 cm^–1^ in Figure 3A and 1352 cm^–1^ in Figure 3B can be attributed to the combination of C–H bending and O–H in-plane bending from the formation of aldehydes and other oxygenated species, as well as a combination of C–O stretching vibrations from the formation of oxygenated species.^20,51^ This growth in positive absorption bands is consistent with the mass gain observed in the gravimetric data for 4BBA and 4IC, confirming the photoinduced oxidation of the samples.

**Scheme 1:**
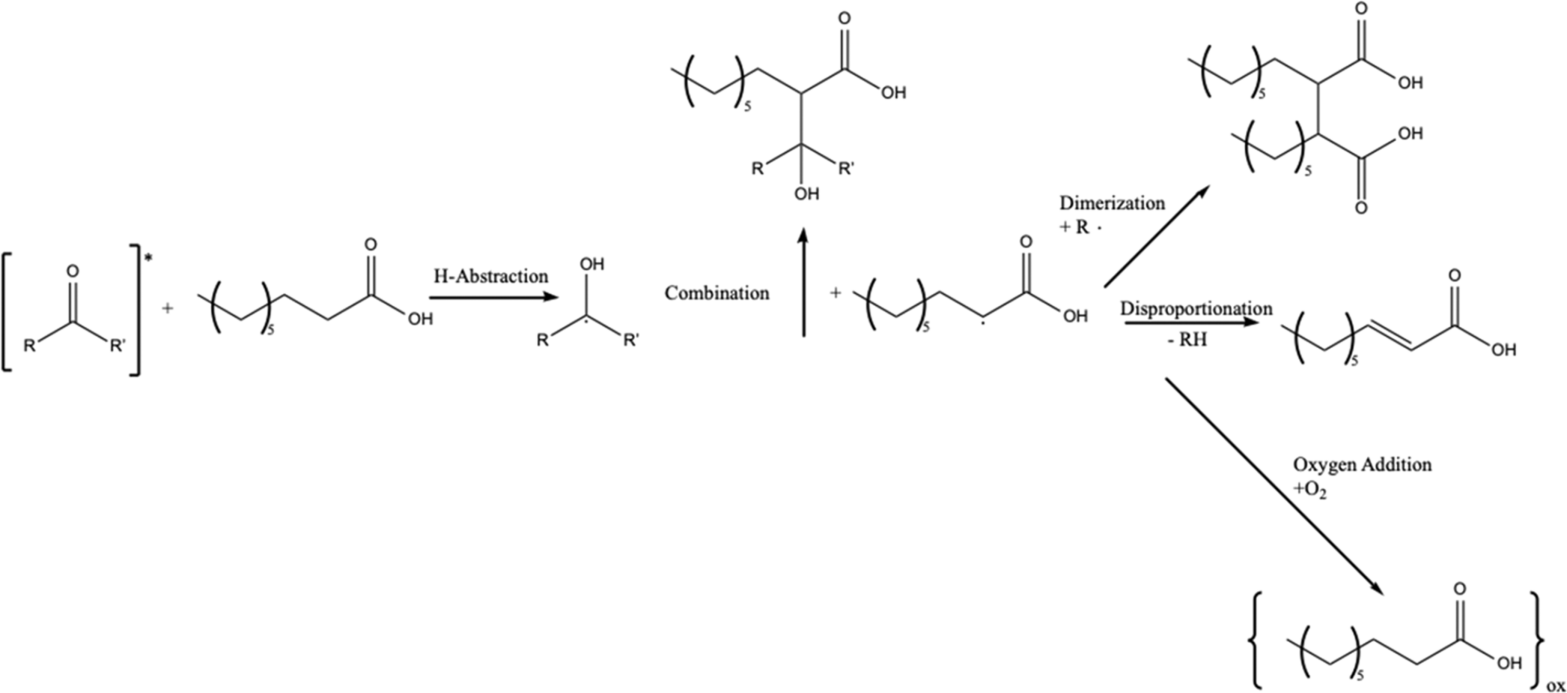
Proposed Mechanism for the Photosensitized Oxidation of NA Based on mechanism proposed by Tinel et al.,^27^ adapted for thin films and the absence of water. The subscript “ox” refers to the reaction products listed in Figure 4.

Both samples containing either 4BBA or 4IC show changes in the absorption intensity in the 2800 to 3000 cm^–1^ region upon exposure to light. These changes, shown in both Figure 3A,B, are due to variations in the C–H symmetric and asymmetric stretching vibrations in NA.^20^ Here, reactions in the thin film, including the oxidation of NA, causes the C–H stretch to shift as the addition of oxygen changes the vibrational modes of NA, leading to the observed positive and negative absorptions in this spectral region. The negative absorptions at 1470 cm^–1^ in Figure 3A and centered at 1474 and 1415 cm^–1^ in Figure 3B can be attributed to the loss of O–H in-plane bending modes of NA due to the formation of dimerization products, including 4BBA and 4IC combination products with NA, as shown in Scheme 1.^20,27^ Finally, the positive absorption band at 1635 cm^–1^ in Figure 3A can be attributed to the C=C stretching mode of unsaturated aldehydes.^20^

**Figure 4:**
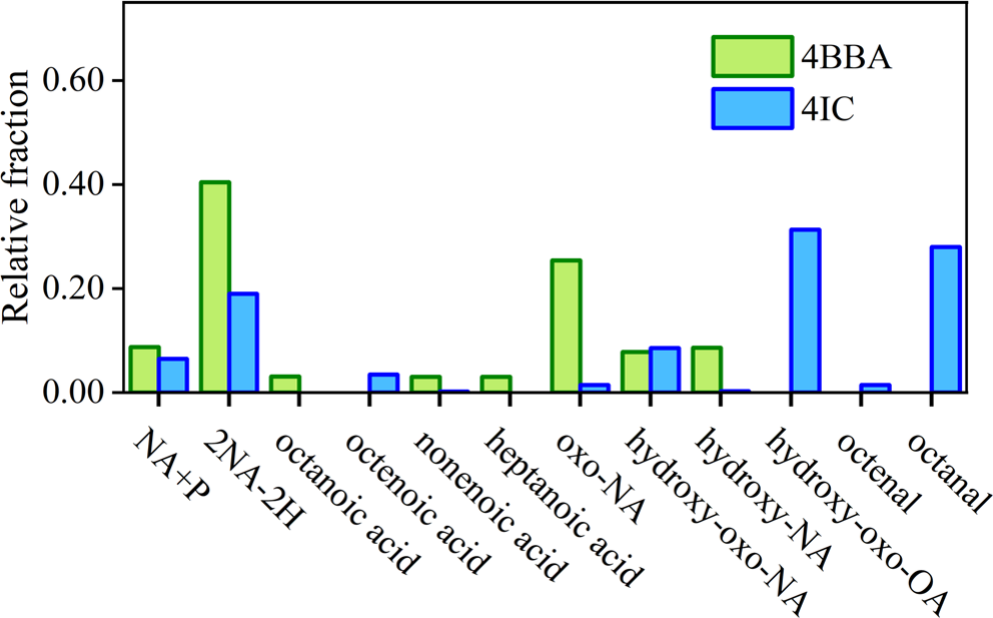
LC-MS relative intensities of products. NA+P represent the dimerization between nonanoic acid and the photosensitizer (either 4BBA or 4IC).

Postreaction LC-MS analysis of irradiated NA thin films containing either 4BBA or 4IC confirms the observations from gravimetric and vibrational spectroscopy analysis. Figure 4 shows that the oxygen addition reaction takes place primarily in NA, while some dimerization of NA (2NA-2H) and combination between the photosensitizer and NA reactions take place. The disproportionation reaction leads to a minor product, nonenoic acid, with both photosensitizers.^51^ These reactions are initiated by the photosensitizer (P) absorbing a photon, leading to a triplet state (P*), as suggested by Tinel et al. for aqueous phase and summarized for thin-films in Scheme 1.^27^ While, these products are not directly detected via gravimetry, as the change in mass is negligible, these reactions can lead to changes in the CH-stretch as shown in the vibrational spectra in Figure 3. Conversely, oxygen addition, with the concomitant increase in mass, leads to a significant number of detected products, such as hydroxy-oxo-NA and hydroxy-NA. Decomposition products of the oxidation reaction were also detected, such as octanoic acid and heptanoic acid, with their oxidation products, such as hydroxy-oxo-octanoic acid (hydroxy-oxo-OA). These products, resulting from oxygen addition, are responsible for mass increases during irradiation observed via QCM and shown in Figure 2. Similar oxidation products were observed for both photosensitizers used, with the major product for 4IC being hydroxy-oxo-OA. For both photosensitizers, the formation of an oxidized fatty acid is consistent with the mass gain observed in the QCM and the increase in O–H and C=O bands observed in the FTIR. For 4BBA, the formation of the dimer product could provide another explanation for the large increase in the C–H stretch observed in the FTIR at 2955 and 2833 cm^–1^.

The light-initiated reaction, shown in Scheme 1 and based on a previously proposed mechanism by Tinel et al.,^27^ shows that the reaction is initiated by the formation of the triplet state of the photosensitizer, leading to free-radical chemistry^4,5,52^
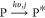
2where *j* is the photochemical kinetic constant. Here, the rate constant for the quenching of P* by nonanoic acid is significantly faster than its relaxation, with the reaction preferentially proceeding to the H-abstraction of NA, as shown in Reaction 3.^27^ The free radicals formed, go on to react with molecular oxygen, forming the oxidized products observed via QCM through multiple pathways
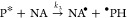
3
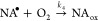
4where NA_ox_ represents the oxidation products that result in oxygen addition and mass increase. As mentioned above, Scheme 1 shows various secondary reactions including dimerization, disproportionation, and combination, with products observed via LCMS (Figure 4). These three secondary reactions have a rate law for the formation of secondary products (SP) that depends on [NA^•^]
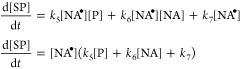
5in which *k*_5_, *k*_6_ and *k*_7_ represent the kinetic constants for the combination, dimerization, and disproportionation reactions, respectively, as shown in Scheme 1. These secondary products ultimately do not contribute to mass increases observed gravimetrically. Thus, the rate of oxygen uptake, summarized in Table 1, is the result of Reaction 4, with a rate law for the production of oxidized nonanoic acid (NA_ox_) shown in eq 6
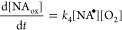
6

Here, a steady-state approximation analysis of NA^•^ leads to an expression of [NA^•^] that depends on the excited state of photosensitizer [P*]
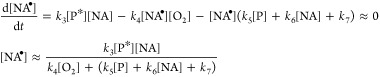
7where the triplet state of the photosensitizer is also an intermediary, with a rate of P* estimated by assuming steady-state conditions
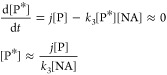
8

Combining eqs 7 and 8 into eq 6 leads to a rate expression that only depends on the amount of NA based on the dimerization secondary reaction. The rate expression is nonlinearly dependent on the amount of photosensitizer ([P]) and the concentration of oxygen ([O_2_]), consistent with the nonlinear increase in the rate of oxygen uptake observed as the amount of photosensitizer increases, as shown in Table 1
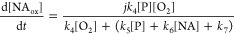
9

Equation 9 suggests that, if secondary reactions are minimized ((*k*_5_[P] + *k*_6_[NA] + *k*_7_) → 0), the oxidation rate becomes more linear and less dependent on the partial pressure of oxygen, with a reduced rate of quenching of P* by nonanoic acid. However, the presence of secondary products and a (*k*_5_[P] + *k*_6_[NA] + *k*_7_) > 0, ultimately leads to a reaction rate of oxygen uptake and mass increase due to oxidation that is not linear with respect to photosensitizer concentration. As shown in Table 1, as the concentration of 4BBA doubles, the reaction rate increases approximately 3-fold. This observation is consistent with *ex situ* LCMS analysis which shows that a significant fraction of the products is those produced during Reaction 5, with the dimerization products being the predominant product. In this case, the increase in rate suggests that the rate constants *k*_4_ and those for secondary reactions are relatively small, leading to a *k*_4_[O_2_] + (*k*_5_[P] + *k*_6_[NA] + *k*_7_) < 1. Conversely, for 4IC, as the concentration of photosensitizer doubles, the reaction rate also roughly doubles, suggesting a nearly linear relationship and thus formation of fewer secondary products, in agreement with LC-MS findings that demonstrate showing smaller fractions of dimer product and higher fractions of oxidation resulting from oxygen addition. As expected from eq 9, an increase in the partial pressure of O_2_ also leads to a nonlinear increase in the rate of mass gain due to oxidation products (see Supporting Information, Figure S2). While the 4BBA/NA thin film, under 60% partial pressure of O_2_, nearly doubles the mass of photooxidation products, the 4IC/NA shows just a slight increase in the oxidation of NA. This low mass increase observed when 4IC is used as the photosensitizer is consistent with the relatively low fraction of secondary reactions in 4IC/NA samples, as shown in Figure 4. Overall, the fewer secondary products observed, with *k*_4_[O_2_] ≫ (*k*_5_[P] + *k*_6_[NA] + *k*_7_), the less dependent on [O_2_] the reaction becomes.

Overall, thin film experiments discussed above show that although the photosensitizer is involved in combination reactions, oxygen uptake in photosensitized oxidation reactions preferentially oxidizes only the fatty acid. The photosensitized oxidation reaction is less dependent on the amount of fatty acid but exhibits a nonlinear dependence on the amount of photosensitizer. This photoinduced oxygen uptake shows a possible mechanism for the aging of aerosols and environmental interfaces that can transform highly hydrophobic components, such as fatty acids, into more complex hydrophilic and oxidized systems. Yet, real environmental interfaces, such as HA and m-DOM, are more complex than the molecular model system, mixing the chromophore with other components that can affect the chemistry in interfaces.

### 3.2 Comparison with Complex Environmental Photosensitizers HA and m-DOM

**Figure 5:**
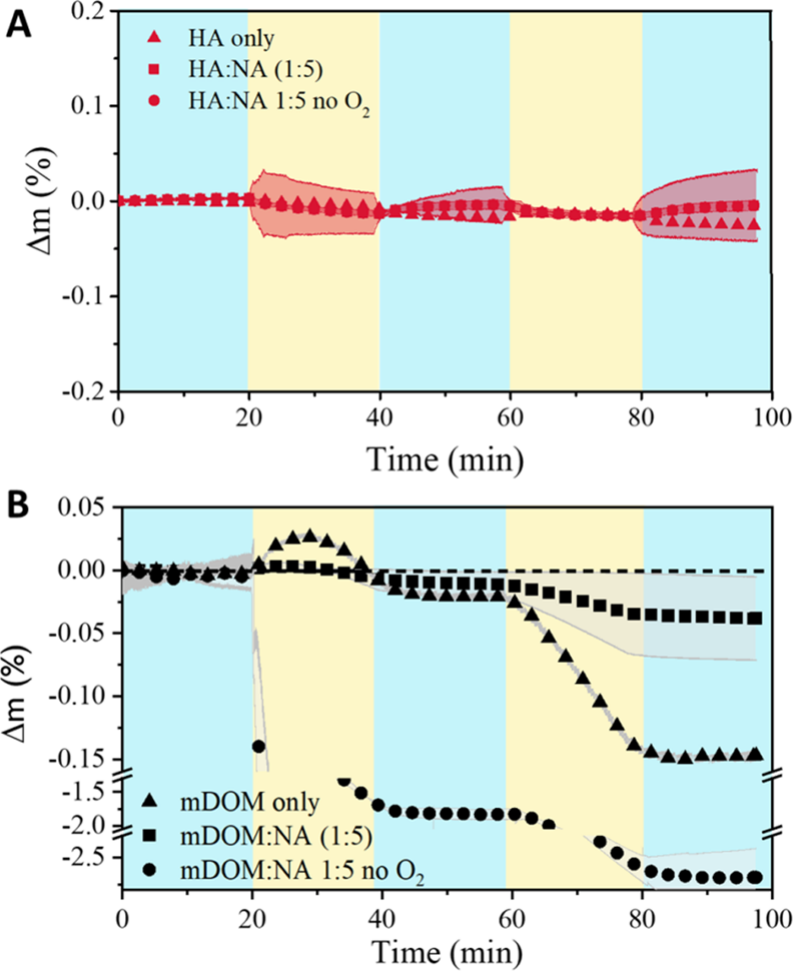
Percentage of mass change in thin films with varying mass ratios of photosensitizer to NA (photosensitizer:NA). Two different environmental photosensitizers were used: (A) humic acid (HA), (B) marine dissolved organic matter (m-DOM). Shade represents standard deviation of triplicate experiments. Only 0.1% of data is plotted for clarity.

Gravimetric irradiation experiments of thin films of NA mixed with either HA or m-DOM under dry air are shown in Figure 5A,B respectively. Similar to Section 3.1, the blue shaded sections represent the periods when the thin film samples were kept in darkness while the yellow shaded sections indicate the periods when samples were exposed to solar simulated light. Figure 5 shows experiments containing either environmental photosensitizers exposed to light/dark cycles without NA, with NA in a 1:5 ratio, and NA in a 1:5 ratio with no oxygen. As shown in Figure 5A, all experiments conducted with HA as photosensitizer show little to no mass change, suggesting no measurable mass gain due to oxidation of NA taking place in the presence of light and HA. A small but measurable loss in mass occurs when HA is exposed to light under dry air.^53^ In the presence of NA, this loss in mass can be compensated with a roughly equivalent mass gain due to light-initiated oxidation, ultimately leading to changes in the gravimetric data that fall within the uncertainty of the QCM.

**Figure 6:**
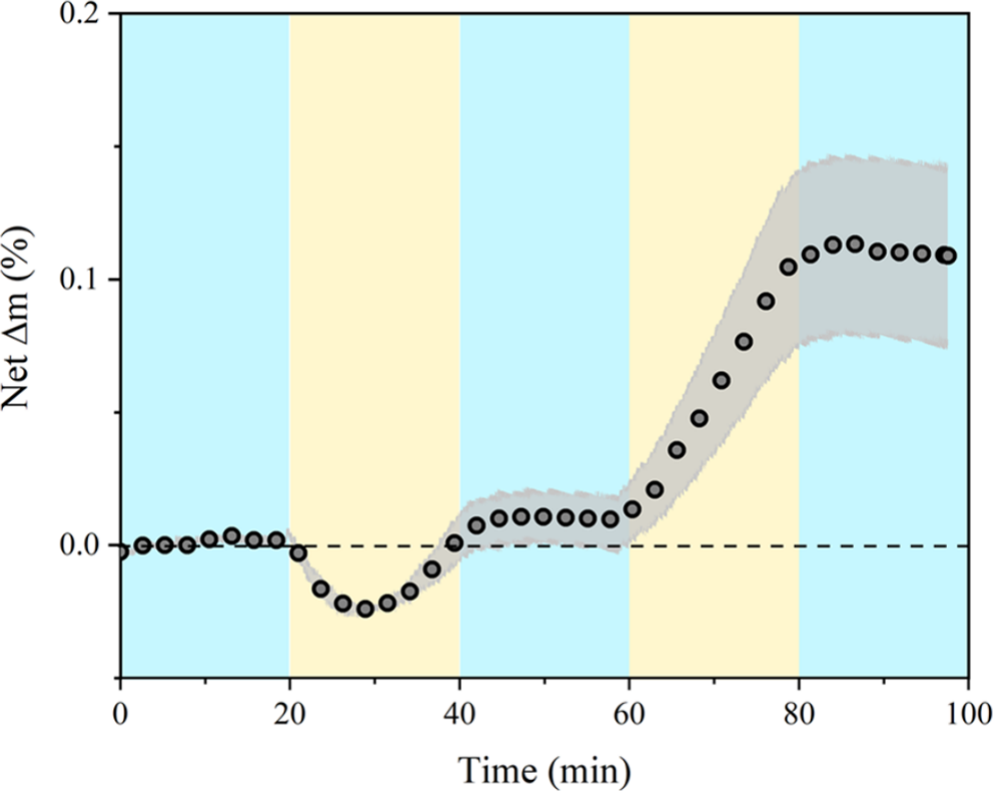
Net mass gain in m-DOM:NA thin film, calculated using eq 10. Shade represents standard deviation of triplicate experiments. Only 0.1% of data is plotted for clarity.

Gravimetric experiments conducted with m-DOM as the photosensitizer are shown in Figure 5B. When a thin film containing a 1:5 ratio of m-DOM/NA in the absence of oxygen was exposed to solar radiation in the QCM, a steep mass loss was observed, totaling in a 2.5% loss in mass at the end of the two 20 min light cycles. This mass loss suggests that m-DOM undergoes fractionation and loss of condensed phase as volatile organic compounds (VOC), which is consistent with photolytic mass loss observed for secondary organic aerosols of similar complexity over longer exposures to solar radiation.^27,53^ In the absence of oxygen, there is no mass gain due to oxidation of NA or m-DOM, making the loss in mass for the thin film m-DOM/NA in the absence of oxygen the largest mass loss observed. Correspondingly, when m-DOM was irradiated in the absence of fatty acid but under dry air, the mass loss decreased substantially, totaling around 0.14% after two 20 min light cycles. Initially, during the first light cycle, the m-DOM thin film undergoes a rapid but small mass increase of about 0.02%. However, after 10 min of irradiation, mass loss became predominant, resulting in a net decrease in mass. We interpret this decrease in the overall rate of mass loss, in part, to the oxidation of m-DOM segments, including nonchromophoric segments of the complex sample.^2,20^ This is supported by vibrational spectroscopy (*vide infra*), where clear absorbance bands suggest the photooxidation of m-DOM. Here, the mass loss due to its fractionation and VOC evolution is counterbalanced by a mass gain due to the reactive uptake of oxygen by m-DOM. Ultimately, the irradiation of thin films containing a 1:5 ratio of m-DOM/NA in the presence of dry air results in less mass loss, indicating that the photooxidation of NA leads to a simultaneous mass gain. As a way to interpret this smaller gain in mass due to photooxidation of the m-DOM/NA 1:5 thin film (net Δ*m*_m–DOM/NA 1:5_), we estimated the net mass gain in the sample as the difference between the oxidations with and without NA
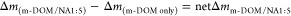
10where Δ*m*_(m–DOM/NA 1:5)_ represents the mass changes in the m-DOM/NA 1:5 thin film, and Δ*m*_(m–DOM only)_ represents the mass changes in m-DOM in the absence of NA. The resulting net change in mass in the m-DOM/NA 1:5 thin film is shown in Figure 6, with a final mass increase of (0.11 ± 0.02)% after two 20 min irradiation cycles.

The initial loss in mass shown in Figure 6 reflects the initial reactive oxygen uptake by the m-DOM thin film during the first light cycle, which reaches a maximum at around 10 min (Figure 5). After that point, both thin films, with and without NA, undergo mass loss, with the sample without NA experiencing a steeper mass loss. Overall, the result is a net gain in mass when NA is present, and suggests that photolytic mass changes in SOA is a complex processed that can be influenced by the surface composition of the aerosol.^15,16,54^ The rate of net mass increases due to oxygen uptake, estimated from the second irradiation cycle in the m-DOM:NA sample in Figure 6, was 3.54 ± 0.01 × 10^–6^ mmol O s^–1^. This rate is lower compared to the photooxidation rates observed when 4IC and 4BBA were used as photosensitizers. Several factors may contribute to this difference. First, not all components in m-DOM are chromophores, leading to a lower effective proportion of photosensitizer to NA in the m-DOM:NA sample.^23^ Second, fractionation and degradation of oxidized m-DOM species can result in a more significant and simultaneous loss in mass. While m-DOM:NA sample shows an initial direct mass increase during the first few minutes of irradiation (Figure 5), similar to that observed for m-DOM alone during the first light cycle, the average mass increase was significantly lower (∼0.01%), suggesting that the reactive uptake of oxygen by NA is slower compared to reactive components within m-DOM.

**Figure 7:**
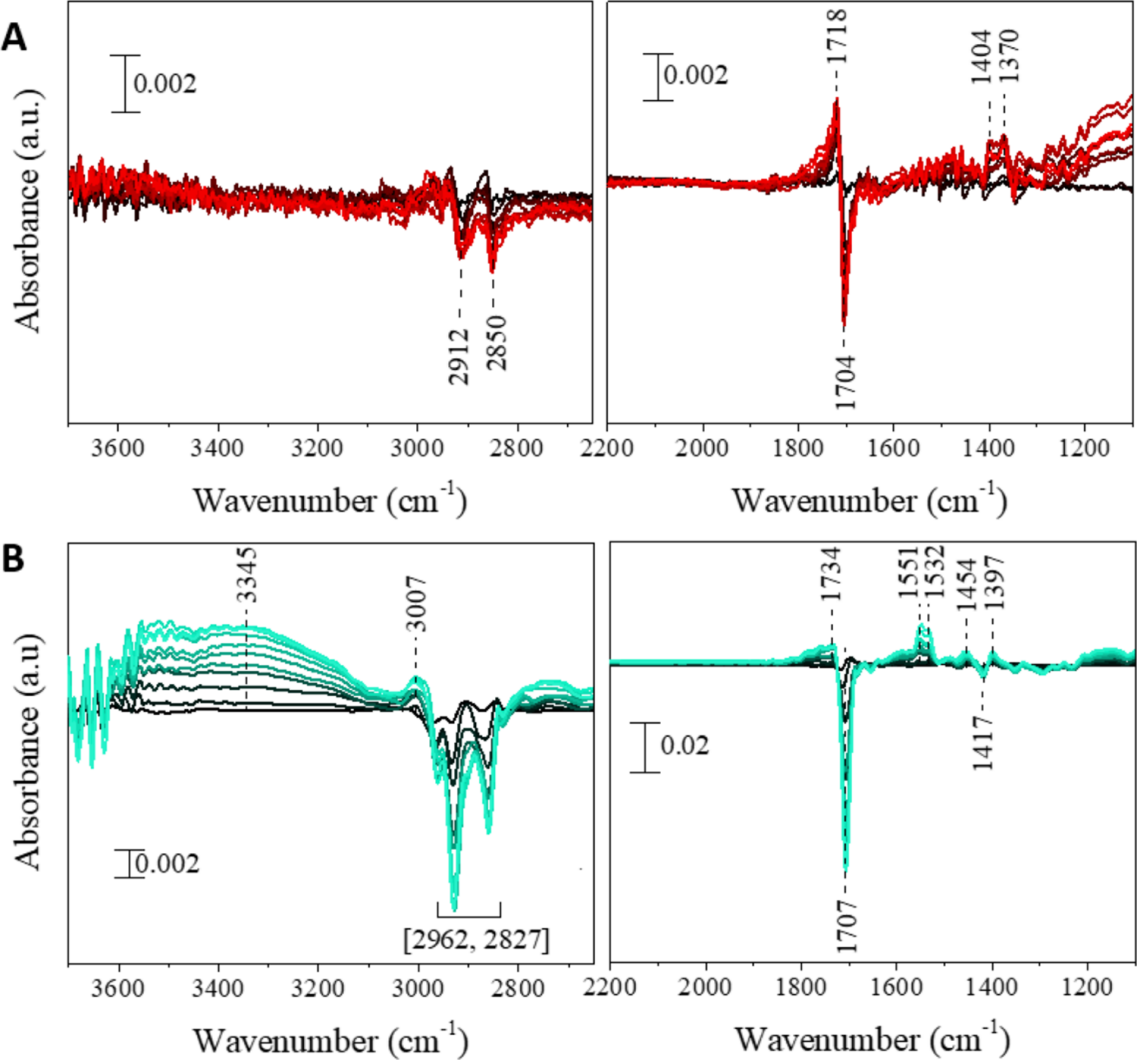
Selected spectra of the ATR–FTIR, referenced to the initial spectrum, of NA in the presence of (A) HA, (B) m-CDOM. Spectra presented with 10 min intervals. Lines become increasingly light with increased time.

The vibrational spectroscopy analysis results for HA, shown in Figure 7A, is consistent with the gravimetric results shown in Figure 5A. Upon exposure to light in the presence of dry air, the 1:5 HA/NA thin film shows no significant changes in absorption in the 3000 to 3600 cm^–1^ region which is consistent with the lack of mass change observed in the QCM experiment. Negative absorption bands at 2912 and 2850 cm^–1^ are likely due to a slight loss of C–H stretch due to fractionation and loss of mass as VOC.^53,55^ This minor mass loss, observed gravimetrically, occurred when HA was exposed to light under dry air in the absence of NA. These possible changes in the HA/NA thin film are also observed as positive absorption band at 1718 cm^–1^, attributed to a growth in the C=O stretching mode for aldehyde and ketone products, likely due to oxidation of NA.^20^ Yet, a simultaneous growth of a negative absorption band at 1704 cm^–1^, also attributed to the stretching mode of C=O functional groups, indicates a loss of mass due to fractionation or decarboxylation of the complex sample, leading to VOC and CO_2_ formation.^33,55,56^ Slight positive absorptions at 1404 and 1370 cm^–1^, corresponding to the bending modes of aldehydic C–H and O–H product functional groups, further support these observations.^20^ These simultaneous processes of oxidation and decarboxylation offset one another, resulting in no measurable mass change in the QCM.

Figure 7B shows the vibrational spectroscopy results of 1:5 m-DOM/NA thin film, where distinct features of oxygenated product formation are identified. The broad positive absorption band at 3345 cm^–1^ is attributed to the O–H stretching from the formation of oxygenated species. This observation is consistent with the gravimetric measurements shown in Figure 5B, in which a lower fraction of mass loss is observed in the sample containing both m-DOM and NA.^20,33,56^ The large negative absorption bands at 2962 and 2827 cm^–1^ are likely due to the loss of C–H stretches due to fractionation and mass loss as VOC. This interpretation is supported by mass loss observed during gravimetric experiments. Figure 7B also shows a sharp negative absorption at 1707 cm^–1^ as a result of the loss of C=O species and decarboxylation within m-CDOM.^20,56^ The slight positive absorption at 1718 cm^–1^ can be attributed the reactive uptake of oxygen leading to the formation of carbonyl functional groups, with the concomitant growth of the C=O stretching mode band for aldehyde and ketone products.^20^ Small but observable absorptions bands between 1550 and 1400 cm^–1^ can be attributed to the bending modes of aldehydic C–H and O–H product functional groups.

Overall, components of m-DOM and HA, including chromophoric and nonchromophoric, undergo photolytic mass loss, which slows down in the presence of oxygen, with the possible formation of reactive oxygen species.^53,57^ This mass loss is only observed in the more complex environmental samples, HA and m-DOM. Notably, when m-DOM is used as a photosensitizer, the mass loss is offset by a mass gain in the presence of NA.^20^ No mass loss is observed using the molecular models 4IC and 4BBA. The complexity of the environmental samples also results in a lower effective ratio between the photosensitizer and the fatty acid, which may explain the differences in reaction rates between m-DOM and the molecular proxies. Overall, 4BBA and 4IC are more effective photosensitizers than m-DOM, producing oxygenated species and unsaturated ketones/aldehydes. This finding is in good agreement with aqueous phase experiments conducted using the m-DOM same sample by Trueblood et al.^20^

## 4 Conclusions

In this work, we estimated the rates of photooxidation of nonanoic acid (NA), a model fatty acid, using molecular photosensitizers as model systems for environmental chromophores. Recent work suggests that marine derived DOM contains more nitrogen organic compounds than their terrestrial counterparts. We compared the potential for initiating photosensitized oxidation of NA using two molecular models—a nitrogen-containing photosensitizer (4IC) and a non-nitrogen photosensitizer (4BBA)—to two complex environmental photosensitizers: a terrestrial humic acid (HA) and a marine dissolved organic matter (m-DOM) system. Gravimetric and vibrational spectroscopy results demonstrate that the oxidation takes place primarily in NA, with 4BBA being the most efficient photosensitizer among those examined, with an increase in mass due to oxygen uptake of 1.5% when the mixture had a 1:5 photosensitizer to NA ratio. Assuming that all the mass is the result of oxygen uptake, the rate of oxygen uptake for mixtures containing 4BBA as photosensitizer was (4.7 ± 0.8 × 10^–5^) mmol O s^–1^. Conversely, similar thin film composition using 4IC as photosensitizer shows a mass increase of up to 0.8% of the initial mass, with a rate of oxygen uptake of (1.5 ± 0.2 × 10^–5^) mmol O s^–1^. The relative effectiveness of 4BBA as a photosensitizer in NA oxidation is due to the higher presence of aromatic species, which has been shown to increase photoactivity, as seen in its more intense absorption bands compared to 4IC.^20,21^ Overall, the rate of photooxidation is dependent on the amount of photosensitizer, and independent of the amount of NA, with differences potentially linked to the optical density of the samples, as 4BBA and 4IC absorb light in different spectral regions. Irradiation of NA in the presence of m-DOM led to a decrease in mass, indicating that the fractionation of m-DOM results in the formation of volatile organic compounds (VOCs). However, the consistently larger mass loss observed when m-DOM was irradiated without NA, along with FTIR spectra, suggests a net mass gain when NA present, although at a slower rate than in the model systems. This overall decrease in mass loss observed when the m-DOM:NA sample is irradiated may involve multiple effects that require further study. HA was found to be a less efficient photosensitizer than 4BBA, 4IC, and m-DOM. While m-DOM and HA show lower photosensitivity activity, the relative abundance of these environmental photosensitizers in both terrestrial and marine boundary layers can lead to higher functionalization and oxidation of aerosol organic fractions.^58−61^

The photosensitized oxidation of NA using 4-benzoylbenzoic acid (4BBA) as a photosensitizer produces leads to the formation of oxo and hydroxy C9, C8 and C7 products, as well as the combination product (4BBA + NA) and the dimerization of NA (2NA-2H). Although the presence of 4IC photooxidation of NA leads to a lower fraction of dimerization, combination, or disproportionation, the products formed upon irradiation of the thin film are similar to those observed with 4BBA. All products formed in the thin films containing either 4BBA or 4IC with NA yield unsaturated and oxidized products analogous to those found in previous experiments using complex environmental photosensitizers.^20,27^ Kinetic analysis suggests that the photooxidation rate of NA is nonlinearly dependent on the amount of photosensitizer. A decrease in photosensitized nonoxidation secondary reactions, such as the combination or dimerization of NA, leads to a rate of reaction becoming more linear with respect to the amount of photosensitizer present in the mixture.

The photooxidation mechanism discussed in this work provides insights on the proportion of hydroxy and hydroxy-oxo fatty acids components within the surface of marine and coastal aerosols.^9,62^ This oxidation process contributes to our understanding of how hydrophobic components, such as fatty acids, influence various aerosol processes, such as hygroscopicity, interface reactivity, and cloud condensation nuclei (CCN) activity of SSA.^37,38,63,64^ Although this study shows lower activity of m-DOM and HA in fatty acid photooxidation compared to molecular models, it also highlights the relevance of such reactions at ocean surfaces and within SSA due to the abundance of organic photosensitizer sources. Given the complexity of environmental chromophores like m-DOM and HA, molecular proxies such as 4BBA and 4IC are essential for molecular-level studies. The results shown in this work provides further insight on the formation of reactive organic species within SSA and how naturally occurring chromophores can influence the pathways and rates of formation for these atmospheric components.^65^

## Data Availability

Data for this study can be accessed in the Center for Aerosol Impacts on Chemistry of the Environment (CAICE) University of California San Diego Library Digital Collections (10.6075/J0KD1Z7Z).

## Supporting Information Available

UV–vis absorbance spectra of 4BBA and 4IC containing NA plotted with the spectral irradiance of the solar simulator. Percentage of mass increase due to photoinduced oxidation of NA in the presence of 4BBA and 4IC at both 20 and 60% O_2_ (PDF)
