# Supplementary material for: Photooxidation of Nonanoic Acid by Molecular and Complex Environmental Photosensitizers
Source: J Phys Chem A. 2024 Nov 5;128(45):9792–803. doi: 10.1021/acs.jpca.4c05608 (PMC11571206; doi:10.1021/acs.jpca.4c05608)
Supplement: Supplementary file 1 — jp4c05608_si_001.pdf [file jp4c05608_si_001.pdf]

Supporting Information:

Photooxidation of Nonanoic Acid by Molecular and Complex Environmental Photosensitizers

Grace A. Freeman-Gallant†, Emily Davis†, Elizabeth Scholer, Onita Alija, and Juan G. Navea*

Chemistry Department, Skidmore College, Saratoga Springs, NY, 12866-1632

† Authors contributed equally to the work

* Corresponding author: Juan G. Navea, jnavea@skidmore.edu

The absorbance spectra of the NA thin films containing molecular photosensitizers 4BBA or 4IC is shown in Figure 1S. While 4BBA absorbance bands are more intense, 4IC absorbance has a larger overlap with the spectral irradiance of the solar simulator.

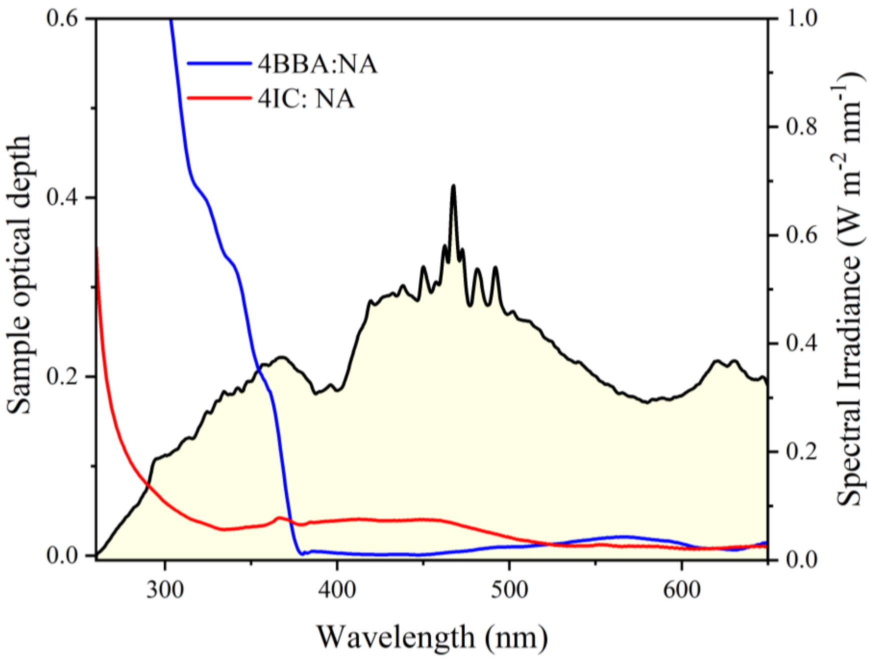

Figure S1: Spectral irradiance of the solar simulator (black line and right axis) in comparison to the absorbance spectra (left axis) of the 4BBA:NA thin film (blue line) and 4IC:NA thin film (red line. All thin film samples were prepared at a 1:5 proportion in mass.

Photooxidation of NA was investigated under dry air (21% O_2) and at an increased partial pressure of approximately 62% O_2. All thin films exposed to light under an enriched O_2 partial pressure where 1:10 mixtures (photosensitizer:NA) to avoid saturation of the QCM, which has a dynamic range up to 2% mass change.

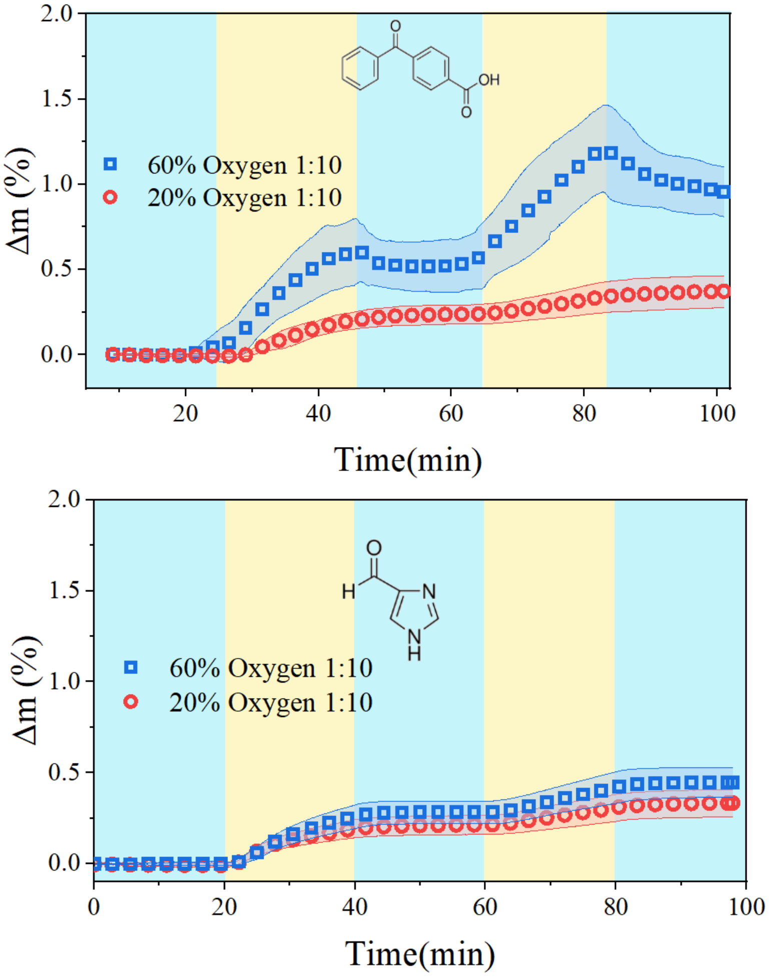

Figure S2: Percentage of mass increase due to photo-induced oxidation of NA in the presence of (A) 4BBA, (B) 4IC, at different partial pressures of O_2. Shade represents standard deviation of triplicate experiments. Only 5% of data is plotted for clarity.

A loss in mass in the absence of light, observed in experiments carried out with 4BBA, is likely due to the loss of highly oxidized products, carried by the dry air flow away from the QCM crystal.
